# Time trends in statin use and incidence of recurrent cardiovascular events in secondary prevention between 1999 and 2013: a registry-based study

**DOI:** 10.1186/s12872-018-0941-y

**Published:** 2018-11-06

**Authors:** Nele Laleman, Séverine Henrard, Marjan van den Akker, Geert Goderis, Frank Buntinx, Gijs Van Pottelbergh, Bert Vaes

**Affiliations:** 1 Department of Public Health and Primary Care, Universiteit Leuven (KU Leuven), Kapucijnenvoer 33, Blok J, 3000 Leuven, Belgium; 2 Institute of Health and Society, Université catholique de Louvain (UCL), Brussels, Belgium; 3 Department of Family Medicine, Maastricht University, Maastricht, The Netherlands

**Keywords:** Statins, Secondary prevention, Cardiovascular diseases, Trends

## Abstract

**Background:**

The current study evaluated time trends of statin use and incidence of recurrent CVD in secondary prevention from 1999 to 2013 and investigated which factors were associated with statin use in secondary prevention.

**Methods:**

Intego is a primary care registration network with 111 general practitioners working in 48 practices in Flanders, Belgium. This retrospective registry-based study included patients aged 50 years or older with a history of CVD. The time trends of statin use and incidence of recurrent CVD in secondary prevention were determined by using a joinpoint regression analysis. Multivariable mixed-effect logistic regression analysis was used to assess factors associated with statin use in patients in secondary prevention in 2013.

**Results:**

The overall prevalence of statin use increased and showed two trends: a sharp increase from 1999 to 2005 (annual percentage change (APC) 25.4%) and a weaker increase from 2005 to 2013 (APC 3.7%). The average increase in statin use was the highest in patients aged 80 and older. Patients aged 70–79 years received the most statins. Men used more statins than women did, but both genders showed similar time trends. The incidence of CVD decreased by an average APC of 3.9%. There were no differences between men and women and between different age groups. A significant decrease was only observed in older patients without statins prescribed. In 2013, 61% of the patients in secondary prevention did not receive a statin. The absence of other secondary preventive medication was strongly associated with less statin use. Gender, age and comorbidity were associated with statin use to a lesser degree.

**Conclusions:**

The prevalence of statin use in secondary prevention increased strongly from 1999 to 2013. Less than 50% of patients with a history of CVD received a statin in 2013. Especially patients who did not receive other secondary preventive medication were more likely to not receive a statin. Despite the strong increase in statin use, there was only a small decrease in the incidence of recurrent CVD, and this occurred mainly in older patients without statins prescribed.

## Introduction

Cardiovascular diseases (CVD) are still the leading cause of death in Europe: in 2012 they were responsible for 47% of all deaths [1, 2]. In the last few decades survival rates after CVD have increased and cardiovascular mortality has decreased [1, 2]. However, individuals with established CVD have a high risk of a recurrent event [2]. The currently recommended pharmacological intervention in secondary prevention is a combination of antithrombotic therapy, statins and, in some cases, antihypertensive agents, independent of age and gender [3–5].

Statins have proven to reduce the risk of recurrent CVD and of cardiovascular and total mortality in secondary prevention [3, 4, 6–8]. Nevertheless, there remains a large gap between current recommendations and clinical practice: previous studies reported that only a minority of patients with a history of CVD receives statins [9–31]. However, to date, no study described time trends of statin use in secondary prevention in combination with the evolution of recurrent CVD in the general population. Although statin non-use is very prevalent, the current study hypothesized that the use of statins in secondary prevention has increased substantially and that this possibly coincides with a lower incidence of recurrent CVD. Furthermore, factors associated with less statin use such as female gender, older age, comorbidity (diabetes, heart failure), smoking, low cholesterol level, longer time after diagnosis and type of CVD have been described [9, 10, 12, 15, 23, 26, 32–36]. However, a recent evaluation of statin use in secondary prevention and a description of factors related to statin use in a real world population are still lacking.

Therefore, the first aim of this retrospective registry-based study was to evaluate time trends of statin use and incidence of recurrent CVD in secondary prevention from 1999 to 2013. The second aim was to investigate which factors are associated with statin use in secondary prevention.

## Methods

### Study design and study population

Data were obtained from Intego, a general-practice-based morbidity registration network at the Department of Public Health and Primary Care of the University of Leuven, Belgium [37]. The Intego procedures were approved by the ethical review board of the Medical School of the University of Leuven (N° ML 1723) and by the Belgian Privacy Commission (no SCSZG/13/079). In 2013, 111 general practitioners (GPs), all using the medical software Medidoc®, collaborated in the Intego project. They worked in 48 practices evenly spread over Flanders, the Northern part of Belgium. GPs applied for inclusion in the registry. Before acceptance of their data, registration performance was audited using a number of algorithms that compared their results with those of all other applicants. Only the data of the practices with an optimal registration performance were included in the database. The Intego GPs prospectively and routinely registered all new diagnoses together with new drug prescriptions, laboratory test results and some background information (including gender and year of birth), using computer-generated keywords internally linked to codes. New data were coded and collected from the GPs’ personal computers with specially framed extraction software and entered into a central database. Registered data were continuously updated and historically accumulated for each patient. New diagnoses were classified according to a very detailed thesaurus automatically linked to the International Classification of Primary Care (ICPC-2). Drugs were classified according to the WHO’s Anatomical Therapeutic Chemical (ATC) classification system.

The current study is a retrospective cohort study that used Intego data from January 1st 1999 to December 31st 2013. In every yearly contact group (patients in contact with their GP for either reason during a year) between 1999 and 2013, we selected all patients aged 50 years or older with a history of CVD (myocardial infarction (MI) (K75), stroke (K90), transient ischemic attack (TIA) (K89), ischemic heart disease (IHD) with and without angina (K74 and K76, respectively) and peripheral arterial disease (PAD) (K92)). Recurrent CVD was defined as an incident case of MI, stroke or TIA in all selected patients, or an incident diagnosis of IHD or PAD in patients without a previous diagnosis of IHD or PAD. MI and stroke were considered as major events. TIA, IHD and PAD were considered as minor events. The prevalence of statin (ATC code C10AA) use (at least 2 prescriptions in the selected year) was registered for each year.

### Clinical characteristics

#### Comorbidity

The medical history of all patients in secondary prevention in 1999 and in 2013 was registered. Besides the history of CVD as defined above, other relevant comorbidities were registered, such as atrial fibrillation, hypercholesterolemia, hypertension and mental disorders (anxiety, depression or overstrain). Furthermore, comorbidities were registered in order to construct the modified Charlson Comorbidity Index (mCCI) [38, 39]. For the presence of renal insufficiency, the glomerular filtration rate (GFR) was estimated (MDRD equation) based on the last creatinine measurement in the 2 years before 1999 or 2013. Whether or not LDL (low-density lipoprotein) had been measured in the 3 years before 1999 or 2013 was registered for all patients included.

#### Pharmacotherapy

The prescription of cardiovascular medication was registered for all patients in secondary prevention in 1999 and in 2013. Data were collected on the prescription of aspirin (ATC code B01AC06), agents acting on the renin-angiotensin system (RAS) (ATC code C09), non-RAS antihypertensive agents (ATC codes C03, C07 and C08) and other lipid lowering medication (LLM) (ATC code C10 except C10AA). Medication use in a specific year was considered positive when at least two prescriptions had been made in that year.

### Statistical analysis

To analyse time trends in age-standardized rates between 1999 and 2013, a joinpoint regression analysis was performed [40]. A joinpoint is a point in the trend curve where a statistically significant change in trend over time is observed. A minimum number of 3 observations from a joinpoint to either end of the data, and a minimum number of 4 observations between two joinpoints were required. The age-standardized rates were computed taken the Flemish population in Belgium as the standard population, using 10-year age groups until 79 years, and 80 years and older as the last age group for standardization. The reference year for the standard population was 1999. From the joinpoint regression model, the annual percentage change (APC) and the average annual percentage change (AAPC) were extracted. APC is calculated for each significant trend from a piecewise log-linear model on the logarithm of the age-standardised rate versus the year. AAPC represents the average of APC estimates per significant trend weighted by the corresponding trend length (number of years in the trend). The trend analysis using the joinpoint regression model was performed using the SEER*Stat software (Joinpoint Trend Analysis software from the Surveillance Research Program of the US National Cancer Institute (available at http://surveillance.cancer.gov/joinpoint)).

Continuous data were summarized using median [P_25_; P_75_], and categorical data using proportions. Factors associated with statin use in patients in secondary prevention in 2013 were assessed using mixed effects logistic regression, which belongs to generalized linear mixed models (GLMM). Each GP practice was treated as a random effect to explore the variability between GP practices in addition to individual patient observations themselves, by age group (50–59 years, 60–79 years, 80+ years). The log-likelihood was estimated using the Laplace approximation. All variables in univariate analysis were candidate for the multivariable model. A stepwise approach was then used to select the best multivariable model. The goodness of fit of the model was assessed using the Akaike information criteria (AIC), Hosmer-Lemeshow test, c-index of the receiver operating characteristic (ROC) curve and the Somers’ D_xy_ rank correlation. The c-index assesses the predictive performance of the model and the Somers’ D_xy_ is an estimate of the rank correlation of the observed binary outcome and the predicted probabilities. In addition, at each step, the maximum variance inflation factor value associated with each parameter in the model was required to be less than 10 for the model to be chosen. A two-sided *p*-value < 0.05 was considered statistically significant. Statistical analyses of the factors associated with statin use in patients in secondary prevention were performed using R Software Version 3.0.3 (Free Software Foundation Inc., Boston, MA, USA) (lme4, pgirmess, gof and ROCR packages) [41].

## Results

**Fig. 1:**
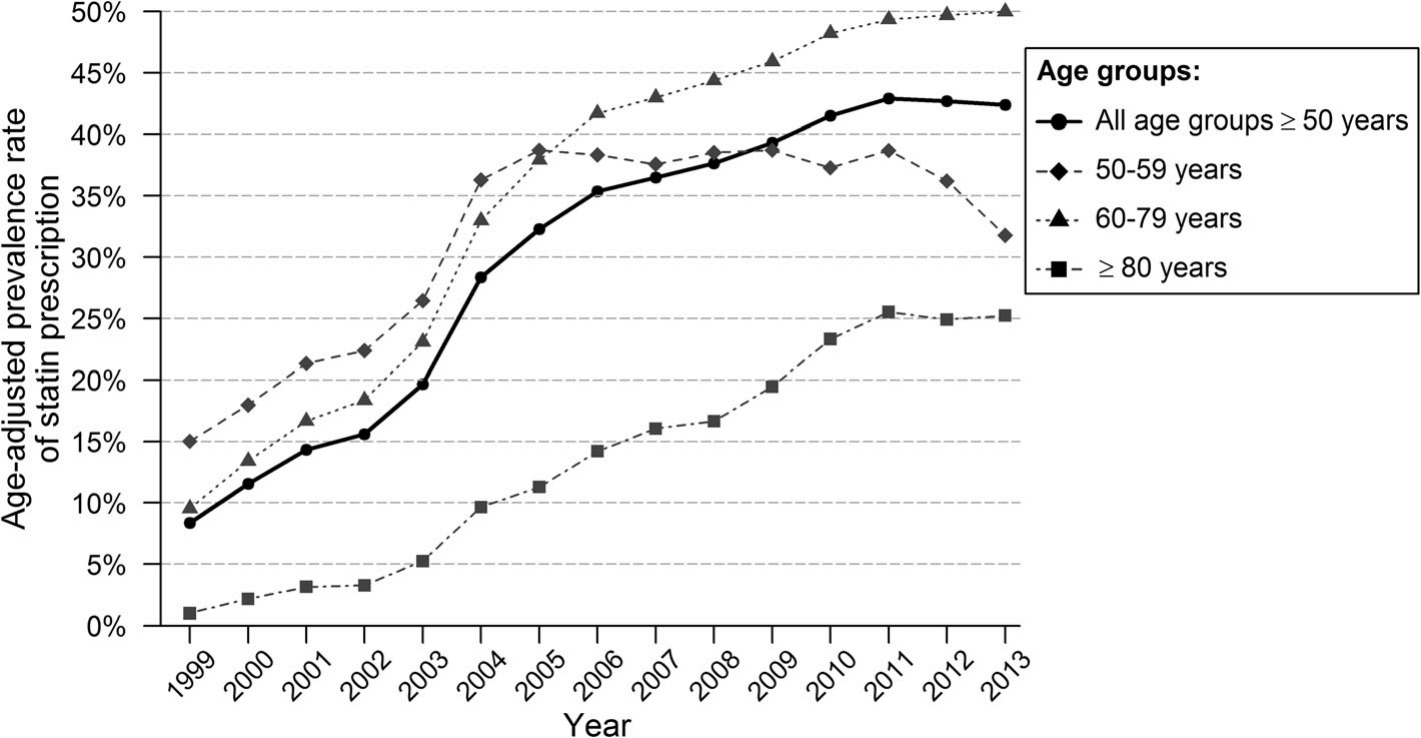
Age-standardized prevalence of statin prescription for patients in secondary prevention, by age group

The age-standardized prevalence rate of statin prescription in secondary prevention increased from 8.4% in 1999 to 39.3% in 2013. Table 1 shows the trends in the prevalence of statin use between 1999 and 2013. Two trends were apparent: there was a sharp increase from 1999 to 2005 and a weaker, but also significant increase from 2005 to 2013 (APC 25.4% (95% CI 21.8–29.0) and 3.7% (95% CI 1.7–5.6), respectively). Figure 1 shows the age-standardized prevalence rate of statin prescription in secondary prevention according to age groups. The average increase in statin prescription was the highest in the oldest age group (AAPC 23.2% and 28.0% in women and men, respectively) and differed significantly with other age groups. In 2013, people aged 70–79 years most often received statins (51%). While patients aged 69 years or younger reached a plateau after 2005, the statin use of patients aged 70 years or older increased further, but less strongly than before. Men used more statins in secondary prevention than women did, but the time trends were similar in both genders (AAPC 12.3% and 12.6%, respectively, AAPC difference − 0.3% (95% CI -2.6; 2.0)).

**Table 1:** Age-standardized prevalence rate of statin use in secondary prevention

Group	ASPR of statin use in 1999	Summary	Trend 1		Trend2		Trend3	
		AAPC	Years	APC	Years	APC	Years	APC
Total	8.4%	12.5 [10.9; 14.1]^*^	1999–2005	25.4 [21.8; 29.0]^*^	2005–2013	3.7 [1.7; 5.6]^*^		
Women	6.5%	12.6 [10.9; 14.3]^*^	1999–2005	25.0 [21.3; 28.8]^*^	2005–2013	4.1 [2.1; 6.2]^*^		
50–59	7.6%	8.3 [5.7; 10.8]^*^	1999–2005	21.0 [15.5; 26.9]^*^	2005–2013	−0.4 [− 3.4; 2.6]		
60–69	13.6%	7.3 [5.1; 9.6]^*^	1999–2006	15.2 [11.4; 19.2]^*^	2006–2013	−0.1 [− 3.4; 3.3]		
70–79	6.6%	14.0 [12.1; 16.0]^*^	1999–2004	31.0 [27.7; 34.4]^*^	2004–2008	11.2 [5.0; 17.8]^*^	2008–2013	1.3 [− 1.3; 3.9]
80+	1.2%	23.2 [17.3; 29.4]^*^	1999–2005	40.0 [26.8; 54.5]^*^	2005–2013	11.9 [5.0; 19.3]^*^		
Men	10.0%	12.3 [10.7; 13.9]^*^	1999–2005	25.4 [21.8; 29.0]^*^	2005–2013	3.4 [1.5; 5.3]^*^		
50–59	18.3%	6.5 [4.7; 8.4]^*^	1999–2005	17.2 [13.2; 21.4]^*^	2005–2013	−0.8 [− 3.0; 1.5]		
60–69	12.7%	10.7 [9.0; 12.4]^*^	1999–2005	24.9 [21.2; 28.8]^*^	2005–2013	1.0 [− 0.9; 3.1]		
70–79	7.6%	15.5 [13.7; 17.4]^*^	1999–2005	29.2 [25.1; 33.3]^*^	2005–2013	6.2 [4.1; 8.4]^*^		
80+	0.8%	28.0 [22.8; 33.3]^*^	1999–2005	59.1 [46.5; 72.9]^*^	2005–2013	8.7 [3.0; 14.6]^*^		

^*^p < 0.05; *ASPR* age-standardized prevalence rate, *AAPC* average annual percentage change, *APC* annual percentage change

**Fig. 2:**
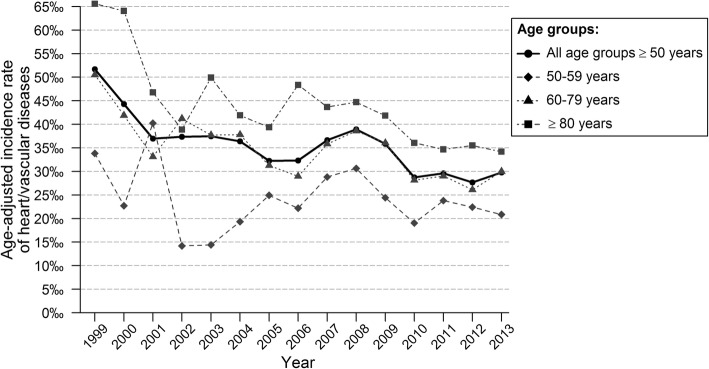
Age-standardized incidence rate of recurrent event for patients in secondary prevention, by age group

**Fig. 3:**
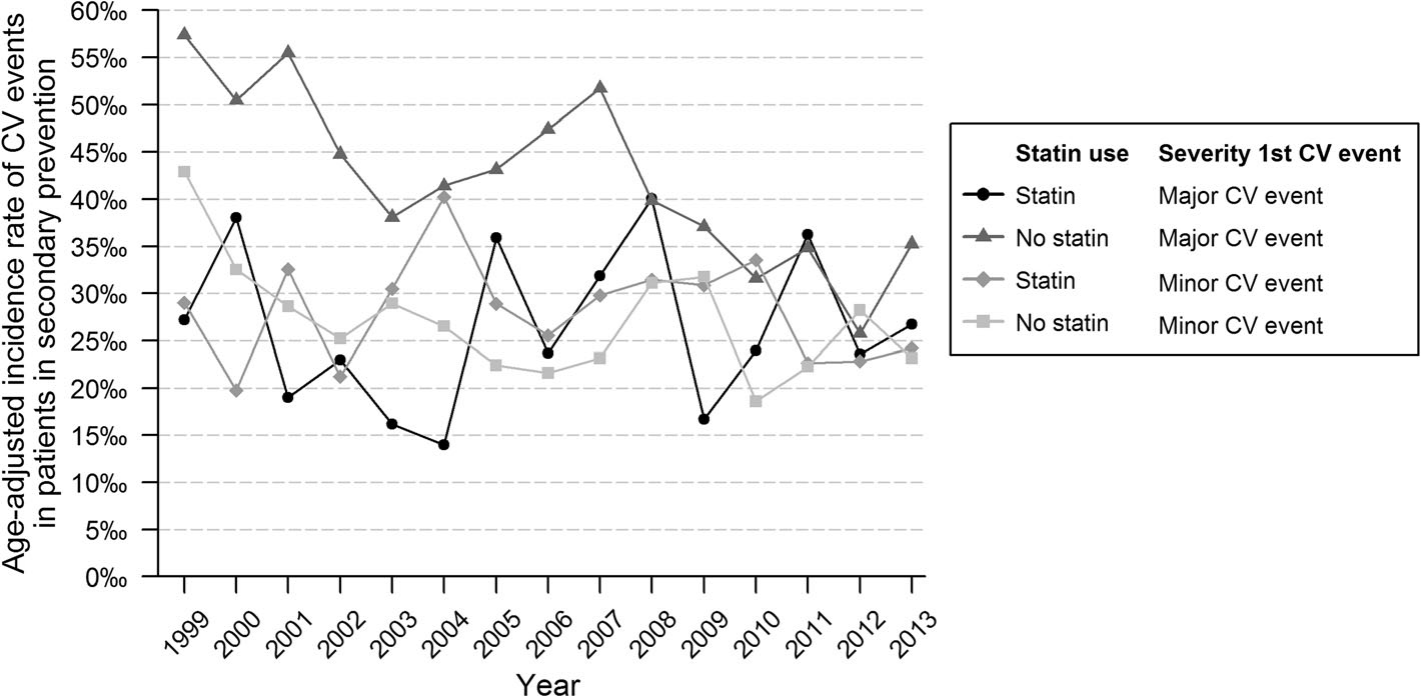
Age-standardized incidence rate of recurrent event for patient in secondary prevention, by statin use or non-use and severity of the first event

Figure 2 and Table 2 show the time trends for the age-standardized incidence rate of recurrent CVD. The incidence was 52/1000 patient years in 1999, it did not show a significant trend until 2001, after which a significant decrease was observed (APC -2.2% (95% CI -3.8; − 0.6)). There were no significant differences in AAPC between men and women and between different age groups. Figure 3 shows the age-standardized incidence rate of recurrent CVD in patients receiving statins and in those without. The incidence rate of recurrent CVD in 1999 was higher in patients without statin prescription (57/1000 patient years vs 27/1000 patient years). A significant decrease of recurrent CVD was seen in patients without statin prescription (AAPC -3.9% (95% CI -5.6; − 2.2) and − 2.5% (95% CI -4.8; − 0.1), in patients with a major or minor first event, respectively). In patients receiving statins no significant trend was observed (AAPC 0.9% (95% CI -3.4; 5.4) and − 0.3% (95% CI -2.9; 2.4) in patients with a major or minor first event, respectively). The decrease in recurrent CVD was mainly seen in older patients (≥60 years) without statin prescription (Table 3).

**Table 2:** Age-standardized incidence rate of CV/heart diseases in secondary prevention

Group	ASIR of CV/heart disease in 1999	Summary	Trend 1		Trend2	
		AAPC	Years	APC	Years	APC
Total	51.7‰	−3.9 [− 7.4; −0.4]*	1999–2001	−13.7 [− 34.4; 13.5]	2001–2013	−2.2 [− 3.8; − 0.6]*
Women	50.3‰	−2.8 [− 4.5; − 1.1]*	1999–2013	−2.8 [− 4.5; − 1.1]*		
50–59	34.5‰	−1.2 [− 6.5; 4.4]	1999–2013^a^	−1.2 [− 6.5; 4.4]		
60–69	33.2‰	− 0.7 [− 3.8; 2.5]	1999–2013	− 0.7 [− 3.8; 2.5]		
70–79	50.5‰	−3.3 [− 6.7; 0.1]	1999–2013	−3.3 [− 6.7; 0.1]		
80+	66.6‰	−3.7 [−5.4; − 2.1]*	1999–2013	−3.7 [− 5.4; − 2.1]*		
Men	52.8‰	−3.3 [− 5.1; − 1.5]*	1999–2013	−3.3 [− 5.1; − 1.5]*		
50–59	33.5‰	−1.1 [− 4.9; 2.9]	1999–2013	− 1.1 [− 4.9; 2.9]		
60–69	51.4‰	− 3.3 [− 5.4; − 1.1]*	1999–2013	− 3.3 [− 5.4; − 1.1]*		
70–79	58.0‰	−3.7 [− 6.3; − 1.1]*	1999–2013	−3.7 [− 6.3; − 1.1]*		
80+	64.1‰	−3.2 [− 5.5; − 0.8]*	1999–2013	−3.2 [− 5.5; − 0.8]*		

^*^p < 0.05; ^a^without 2002 because it was 0, *ASIR* age-standardized incidence rate, *AAPC* average annual percentage change, *APC* annual percentage change

**Table 3:** Age-standardized incidence rate of CV/heart diseases in secondary prevention among statin users and statin non-users

Group	ASIR of CV/heart disease in 1999	Summary	Trend 1		Trend2	
		AAPC	Years	APC	Years	APC
Total	51.7‰	−3.9 [− 7.4; − 0.4]*	1999–2001	− 13.7 [− 34.4; 13.5]	2001–2013	−2.2 [− 3.8; − 0.6]*
Statin users	69.5‰	−3.4 [− 6.7; 0.1]	1999–2001	− 34.5 [− 62.6; 14.7]	2001–2013	−0.4 [− 3.7; 2.9]
50–59^a^	28.3‰^a^	2.6 [−4.3; 9.9]	2001–2013	2.6 [− 4.3; 9.9]		
60–79	37.3‰	−0.5 [− 2.8; 1.9]	1999–2013	−0.5 [− 2.8; 1.9]		
80 + ^b^	55.9‰^b^	−7.2 [− 29.0; 21.3]	2004–2013	−7.2 [− 29.0; 21.3]		
Statin non-users	52.6‰	−3.2 [−4.4; − 2.0]*	1999–2001	− 14.1 [− 33.8; 11.6]	2001–2013	− 2.3 [− 3.8; − 0.8]*
50–59	37.3‰	−1.8 [−6.7; 3.3]	1999–2013	− 1.8 [− 6.7; 3.3]		
60–79	51.7‰	−3.2 [− 4.6; − 1.7]*	1999–2013	−3.2 [− 4.6; − 1.7]*		
80+	64.2‰	−3.4 [− 5.0; − 1.8]*	1999–2013	−3.4 [− 5.0; − 1.8]*		

^*^p < 0.05, ^a^data for years 1999 to 2000 were excluded because of the small sample size (*N* < 100), ^b^data for years 1999 to 2003 were excluded because of the small sample size (N < 100), *ASIR* age-standardized incidence rate, *AAPC* average annual percentage change, *APC* annual percentage change

The first part of Table 4 shows the main characteristics of patients in secondary prevention in 1999 and in 2013. Mean age, gender distribution and prevalence of different CVD were similar. The crude prevalence of statin use in secondary prevention increased sharply from 7.4% in 1999 to 39.3% in 2013. In 2013, more people received other secondary preventive medications (aspirin, RAS- and non-RAS-antihypertensive agents) than in 1999. In terms of comorbidity, there was an increase of hypercholesterolemia, hypertension, COPD, diabetes and cancer between 1999 and 2013.

**Table 4:** Characteristics of patients on secondary prevention

Variables	1999	2013	Age group in 2013								
	Total (*n* = 4007)	Total (*n* = 6295)	50–59 years (*n* = 1077)			60–79 years (*n* = 3211)			80+ years (*n* = 2077)		
	*n* (%) or Median [P25; P75]	n (%) or Median [P25; P75]	*n* (%) or Median [P25; P75]	Statin use cat.^a^ %	Statin use ref. cat.^b^ %	*n* (%) or Median [P25; P75]	Statin use cat.^a^ %	Statin use ref. cat.^b^ %	n (%) or Median [P25; P75]	Statin use cat.^a^ %	Statin use ref. cat.^b^ %
Statin use, *n* (%)	295 (7.4)	2477 (39.3)	330 (32.8)			1614 (50.3)			533 (25.7)		
Baseline characteristics											
Age (years), median [IQR]	73 [66; 79]	74 [64; 82]	55 [52; 57]			70 [65; 75]			85 [82; 89]		
Women, *n* (%)	1839 (45.9)	2871 (45.6)	448 (44.5)	24.1^***^	39.7	1280 (39.9)	41.6^***^	56.0	1143 (55)	20.4^***^	32.1
LDL measurement, *n* (%)	1559 (38.9)	2405 (38.2)	421 (42.0)	23.3^***^	39.6	1040 (32.4)	38.8^***^	55.8	944 (45.5)	14.6^***^	34.9
Type of CVD history											
MI, *n* (%)	848 (21.2)	1507 (23.9)	331 (32.9)	36.3*	31.1	786 (24.5)	59.0^***^	47.4	390 (18.8)	31.0**	24.4
Stroke, *n* (%)	831 (20.7)	1496 (23.8)	248 (24.6)	29.8	33.7	672 (20.9)	46.3^**^	51.3	576 (27.7)	20.5^***^	27.6
TIA, *n* (%)	631 (15.7)	963 (15.3)	67 (6.7)	41.8	32.1	414 (12.9)	49.3	50.4	482 (23.2)	21.8^**^	26.8
IHD without angina, *n* (%)	754 (18.8)	1147 (18.2)	144 (14.3)	42.4^**^	31.2	648 (20.2)	61.3^***^	47.5	355 (17.1)	33.2^***^	24.1
IHD with angina, *n* (%)	1582 (39.5)	1809 (28.7)	173 (17.2)	46.8^***^	29.9	938 (29.2)	54.1^**^	48.7	698 (33.6)	27.5	24.7
PAD, *n* (%)	1011 (25.2)	1669 (26.5)	242 (24.0)	27.3^**^	34.5	873 (27.2)	45.9^**^	51.9	554 (26.7)	27.4	25.0
Comorbidities											
Atrial fibrillation, *n* (%)	430 (10.7)	870 (13.8)	24 (2.4)	45.8	32.5	359 (11.2)	53.8	49.8	487 (23.4)	21.1^**^	27.0
Hypercholesterolemia, *n* (%)	1040 (26.0)	2075 (33.0)	292 (29.0)	50.7^***^	25.5	1134 (35.3)	60.6^***^	44.6	649 (31.2)	34.2^***^	21.8
Hypertension, *n* (%)	1396 (34.8)	2772 (44.0)	344 (34.2)	36.6*	30.8	1422 (44.3)	51.7	49.1	1006 (48.4)	24.8	26.5
Mental disorder, *n* (%)	625 (15.6)	1310 (20.8)	289 (28.7)	32.5	32.9	627 (19.5)	45.1^**^	51.5	394 (19.0)	17.5^***^	27.6
Co-medications											
Aspirin and antihypertensive agents											
Aspirin + RAS and Non-RAS	75 (1.9)	707 (11.2)	88 (8.7)	Ref cat		458 (14.3)	Ref cat		161 (7.8)	Ref cat	
Aspirin + RAS or Non-RAS, *n* (%)	295 (7.4)	1464 (23.3)	171 (17.0)	73.5	80.7	959 (29.9)	73.7^**^	81.0	334 (16.1)	63.0^**^	70.8
Aspirin alone, *n* (%)	68 (1.7)	226 (3.6)	45 (4.5)	68.9	80.7	147 (4.6)	58.5^***^	81.0	34 (1.6)	44.1^***^	70.8
RAS or Non-RAS and no aspirin, *n* (%)	702 (17.5)	1808 (28.7)	256 (25.4)	38.7^***^	80.7	1014 (31.6)	60.4^***^	81.0	538 (25.9)	44.8^***^	70.8
None of these 3 categories, *n* (%)	2942 (73.4)	2797 (44.4)	535 (53.1)	12.7^***^	80.7	1091 (34.0)	16.1^***^	81.0	1171 (56.4)	4.6^***^	70.8
Other lipid-lowering medication	73 (4.3)	237 (3.8)	37 (3.7)	48.6**	32.2	164 (5.1)	43.3*	50.6	36 (1.7)	30.6	25.6
Charlson comorbidity index											
mCCI index^c^, median [IQR]	5 [3; 6]	5 [4; 7]	3 [2; 4]			5 [4; 6]			7 [6; 8]		
MI/ IHD w/o angina, *n* (%)	1395 (34.8)	2364 (37.6)	442 (43.9)	36.4**	29.9	1269 (39.5)	59.3^***^	44.3	653 (31.4)	32.3^***^	22.6
Heart failure, *n* (%)	364 (9.1)	500 (7.9)	20 (2.0)	55.0^**^	32.3	156 (4.9)	59.6^**^	49.8	324 (15.6)	19.1^**^	26.9
PAD, *n* (%)	1011 (25.2)	1669 (26.5)	242 (24.0)	27.3^**^	34.5	873 (27.2)	45.9^**^	51.9	554 (26.7)	27.4	25.0
TIA/stroke, *n* (%)	1333 (33.3)	2283 (36.3)	305 (30.3)	31.8	33.2	1014 (31.6)	46.6^**^	51.9	964 (46.4)	21.1^***^	29.6
Dementia, *n* (%)	94 (2.3)	301 (4.8)	17 (1.7)	23.5	32.9	87 (2.7)	40.2*	50.5	197 (9.5)	14.7^***^	26.8
COPD/asthma, *n* (%)	579 (14.4)	1580 (25.1)	276 (27.4)	31.9	33.1	862 (26.8)	48.4	51.0	442 (21.3)	24.7	25.9
GI ulcer, *n* (%)	471 (11.8)	647 (10.3)	65 (6.5)	30.8	32.9	331 (10.3)	49.5	50.3	251 (12.1)	21.9	26.2
Liver disease, *n* (%)	110 (2.7)	361 (5.7)	52 (5.2)	40.4	32.4	200 (6.2)	48.0	50.4	109 (5.2)	20.2	26.0
Diabetes mellitus, *n* (%)	644 (16.1)	1587 (25.2)	235 (23.3)	38.7^**^	31.0	855 (26.6)	58.9^***^	47.1	497 (23.9)	27.4	25.1
Paralysis, *n* (%)	122 (3.0)	141 (2.2)	18 (1.8)	55.6^**^	32.4	65 (2.0)	43.1	50.4	58 (2.8)	15.5*	26.0
Cancer, *n* (%)	382 (9.5)	1207 (19.2)	191 (19.0)	20.9^***^	35.5	631 (19.7)	43.3^***^	52.0	385 (18.5)	23.4	26.2
Leukaemia, *n* (%)	9 (0.2)	40 (0.6)	10 (1.0)	20.0	32.9	15 (0.5)	46.7	50.3	15 (0.7)	33.3	25.6
Hodgkin’s lymphoma, *n* (%)	12 (0.3)	44 (0.7)	6 (0.6)	16.7	32.9	27 (0.8)	40.7	50.3	11 (0.5)	36.4	25.6
HIV-infection/ AIDS, *n* (%)	NA	NA	NA								
Renal insufficiency^d^, *n* (%)											
Yes vs no	468 (11.7)	665 (10.6)	14 (1.4)	50.0	37.7	192 (6.0)	49.5	54.6	459 (22.1)	25.3^**^	31.9
Not measured vs measured	755 (18.8)	970 (15.4)	214 (21.3)	13.6^***^	38.0	372 (11.6)	19.6^***^	54.3	384 (18.5)	6.0^***^	30.1

*IQR* inter-quartile range, *LDL* low-density lipoprotein, *CVD* cardiovascular disease, *MI* myocardial infarction, *TIA* transient ischemic attack, *IHD* ischemic heart disease, *PAD* peripheral arterial disease, *RAS* renin-angiotensin system, *mCCI* modified Charlson comorbidity index, *COPD* chronic obstructive pulmonary disease, *GI* gastro-intestinal (duodenal or gastric)

^***^p < 0.001, ^**^p < 0.05, ^*^p < 0.10 (p-values are derived from a simple logistic regression model)

^a^Statin use cat.: statin use in the category of interest; ^b^Statin use ref. cat.: statin use in the category of reference; ^c^Missing kidney function is treated as a score of 0; ^d^the last value of the creatinine was taken if it was measured in the last 3 years. The eGFR was computed using the MDRD formula and if eGFR< 45 = > renal insufficiency

In the second part of Table 4, the 2013 study population was split in different age groups (50–59 years, 60–79 years and 80+ years), because important differences in prevalence of statin use were observed (32.8%, 50.3% and 25.7%, respectively). Age groups 60–69 years and 70–79 years were combined because the prevalence of statin use was similar. In every age group, the prevalence of statin use was compared in subjects with and without specific clinical characteristics. In all age groups, women, subjects with an LDL-measurement in the past 3 years, subjects with no registered history of hypercholesterolemia, MI or IHD with angina, and subjects with no GFR estimated or not receiving any other preventive medication, showed a lower prevalence of statin use. People in the oldest age groups with a history of stroke, TIA or dementia were less often prescribed statins than people without these conditions. At younger age (50–79 years), individuals with cancer or PAD were less likely to receive a statin than peers without these conditions, whereas the opposite was observed for patients with diabetes.

Table 5 shows the results of the multivariable mixed-effect logistic regression analysis. In all age groups, the absence of other preventive medication was strongly associated with less statin use: patients who did not receive aspirin, RAS- or non-RAS-antihypertensive agents showed an OR of 0.03 (95% CI 0.01–0.06), 0.04 (95% CI 0.03–0.05) and 0.02 (95% CI 0.01–0.03), in age groups 50–59 years, 60–79 years and 80+, respectively. The prescription of other LLM than statins decreased the odds of having a statin prescribed. Women were less likely to receive statins in secondary prevention than men. In subjects aged 60–79 older age predicted greater statin use (OR 1.25 (95% CI 1.02–1.52)). On the other hand, older age predicted lower statin use in the oldest age group (OR 0.87 (95% CI 0.84–0.90)). In all age groups, hypertension was associated with less statin use, whereas hypercholesterolemia predicted greater statin use. Diabetes was associated with a higher statin use in subjects aged 60–79 years (OR 1.52 (95% CI 1.22–1.88)).

**Table 5:** Determinants of statin use in secondary prevention (mixed-effect logistic regression)

Variables	Multivariable analysis (50–59 years)		Multivariable analysis (60–79 years)		Multivariable analysis (80+ years)	
	OR [95%CI]	*p*-value	OR [95% CI]	*p*-value	OR [95% CI]	*p*-value
Baseline characteristics						
Age, per year increase			1.25 [1.02; 1.52]	0.030	0.87 [0.84; 0.90]	< 0.001
Women	0.59 [0.42; 0.84]	0.003	0.64 [0.54; 0.77]	< 0.001	0.68 [0.51; 0.89]	0.005
LDL measurement			0.64 [0.49; 0.83]	0.001	0.50 [0.37; 0.68]	< 0.001
Type of CVD history						
MI			1.42 [1.15; 1.75]	0.001		
IHD without angina			1.40 [1.12; 1.75]	0.003	1.48 [1.06; 2.09]	0.022
IHD with angina	1.60 [1.04; 2.45]	0.030			1.36 [1.03; 1.80]	0.033
Comorbidities						
Atrial fibrillation					0.66 [0.48; 0.92]	0.013
Hypercholesterolemia	3.59 [2.45; 5.30]	< 0.001	2.40 [1.98; 2.92]	< 0.001	2.93 [2.18; 3.97]	< 0.001
Hypertension	0.65 [0.44; 0.96]	0.030	0.58 [0.48; 0.70]	< 0.001	0.65 [2.18; 3.97]	0.002
Mental disorder					0.63 [0.43; 0.91]	0.015
Co-medications						
Aspirin and antihypertensive agents						
Reference: Aspirin + RAS and	1.00		1.00		1.00	
Non-RAS						
Aspirin + RAS or Non-RAS	0.63 [0.28; 1.35]	0.236	0.61 [0.44; 0.85]	0.004	0.67 [0.40; 1.12]	0.126
Aspirin alone	0.48 [0.19; 1.19]	0.110	0.23 [0.15; 0.36]	< 0.001	0.30 [0.13; 0.71]	0.006
RAS or Non-RAS	0.15 [0.08; 0.28]	< 0.001	0.36 [0.27; 0.47]	< 0.001	0.37 [0.24; 0.57]	< 0.001
None of these 3 categories	0.03 [0.01; 0.06]	< 0.001	0.04 [0.03; 0.05]	< 0.001	0.02 [0.01; 0.03]	< 0.001
Other lipid lowering medication	0.39 [0.17; 0.88]	0.024	0.24 [0.17; 0.35]	< 0.001	0.19 [0.08; 0.42]	< 0.001
Charlson comorbidity index						
mCCI index^a^			0.89 [0.83; 0.95]	< 0.001		
Liver disease					0.54 [0.28; 1.00]	0.054
Diabetes, mellitus			1.52 [1.22; 1.88]	< 0.001		
Paralysis	5.12 [1.51; 17.60]	0.009				
Cancer	0.63 [0.39; 1.01]	0.059				
Renal insufficiency						
Yes	1.88 [0.53; 6.60]	0.318	0.98 [0.65; 1.46]	0.904		
Not measured	0.34 [0.20; 0.55]	< 0.001	0.39 [0.27; 0.55]	< 0.001		

*OR* odds ratio, *CI* confidence interval, *LDL* low-density lipoprotein, *CVD* cardiovascular disease, *MI* myocardial infarction, *IHD* ischemic heart disease, *RAS* renin-angiotensin system, *mCCI* modified Charlson comorbidity index

^a^Missing kidney function is treated as a score of 0

The c-index of the full model was 0.86, 0.84 and 0.90 in the respective age groups (50–59 years, 60–79 years and 80+ years), demonstrating a good total discriminant ability to identify patients with statin use. The c-index of the model that included only co-medication was 0.83, 0.80 and 0.86, respectively. The c-index of the model without co-medication was 0.76, 0.73 and 0.80, respectively. The Somers’ D_xy_ rank correlation of the full model between the predicted probabilities and the observed outcome was 0.72, 0.68 and 0.80, respectively. Finally, the *p*-values associated with the Hosmer and Lemeshow test of the full model were 0.62, 0.10 and 0.73, respectively, demonstrating that the predicted values from the model fit the observed values.

## Discussion

### Major findings

This large registry-based study showed that the prevalence of statin use in secondary prevention increased strongly from 1999 to 2005, and more slightly from 2005 to 2013. Despite the strong increase in statin use in secondary prevention, there was rather a modest decline in the incidence of CVD, and this occurred mainly in older patients without statins prescribed. In 2013, less than 50% of the patients with a history of CVD received a statin. The absence of other secondary preventive medication was strongly associated with less statin use. Gender, age and comorbidity were associated with statin use to a lesser degree.

### Time trends in age-standardized prevalence rate of statin use

Analogous to previous studies, we found that the prevalence of statin use in secondary prevention has increased over the last few decades [9–18, 20, 21, 23–36, 42–44]. The rise in statin use has been linked to guideline changes, promotions by the pharmaceutical industry, media reports, reimbursement conditions, entrance of generic medication and reductions of the price [10, 12, 15, 17, 18, 21, 24, 25, 28, 32, 33, 42, 43, 45].

This study computed AAPC of the prevalence of statin use in secondary prevention, which showed two trends: there was a sharp increase from 1999 to 2005 and a weaker, but also statistical significant increase from 2005 to 2013. This suggests that the steep increase in statin use in secondary prevention in the first time period was mainly linked to the growing literature in support of statins and to guideline changes. After 2005, when statins became less expensive for Belgian patients, the prevalence of statin use in secondary prevention rather seemed to reach a plateau. This finding is in line with previous studies [14, 15, 21, 24, 26, 27, 33, 36].

### Incidence of recurrent CVD

The current study showed that the incidence of recurrent CVD did not decrease much compared to the strong increase in the prevalence of statin use. Possible explanations are a higher prevalence of other cardiovascular risk factors and an inadequate control of other risk factors and perhaps the benefits of statins in most clinical trials do not translate to real world populations, which differ in ways that may adversely affect the risk-benefit balance (more comorbidities, polypharmacy, disability, etc). The current study also showed an increase in prevalence of diabetes, hypertension and hypercholesterolemia between 1999 and 2013. Unfortunately, insufficient data on the body mass index (BMI) and smoking status are available in the Intego registry. However, the Intego registry is representative for the Flemish population and the Belgian Health Interview Survey showed that the prevalence of overweight and smoking status in the age group 55+ years in Flanders did not change considerably from 1997 to 2013 (from 56.3 to 59% and from 17.6 to 16.9%, respectively) [46]. Moreover, the Euroaspire surveys described time trends in lifestyle, risk factor control and use of evidence-based medications in patients with coronary heart disease in Europe [14]. These surveys concluded that there was an increase of obesity and diabetes and that the proportion of smokers and the level of physical activity had remained stable from 1999 to 2013. Other studies showed similar results of cardiovascular risk factor control in secondary prevention [22, 28, 47–49].

The minor decrease of the incidence of recurrent CVD was only observed in patients without statins prescribed. The incidence in this group was higher in 1999 and evolved towards the lower incidence of recurrent CVD in patients with statins prescribed in the 15 years thereafter, to be around 25 per 1000 patient years. However, since this study was only an observational study, both groups cannot be considered identical. Therefore, no conclusions could be drawn about the possible effect of statins on the incidence of recurrent CVD. This study was only designed to observe trends and generate hypotheses. Furthermore, no data were available on other factors that might have led to improved CVD outcomes in the same time period, like a reduction in trans fat consumption or attention to the treatment of sleep apnea.

### Factors associated with statin use

Despite the strong increase in statin use in secondary prevention, the current study confirmed, in line with the results of previous studies, that less than 50% of patients in secondary prevention received a statin in 2013 [9–18, 20, 21, 23–36, 42–44].

The absence of other secondary preventive medications (aspirin, RAS and non-RAS antihypertensive agents) was strongly associated with less statin use in secondary prevention. Possible explanations of these findings could be doctor-related factors such as poor knowledge and application of the guidelines, preference against polypharmacy or the doctor’s reading of the evidence that may be at variance with the guidelines, and patient-related factors such as poor adherence to therapy. Previous studies also found an association between the use of statins, aspirin and antihypertensive agents [11, 23].

Women were less likely to receive statins in secondary prevention than men. This finding is also in line with previous studies [9, 12, 15, 19, 23, 29, 31–34]. Some of these studies showed that women also received less intensive statin therapy [9, 27, 34]. A possible reason for this observation might be the fact that women have a higher risk of statin adverse effects [50]. Furthermore, although statin therapy was shown to be an effective intervention in the secondary prevention of cardiovascular events in women, there was no benefit on stroke and all-cause mortality in women [51].

In the age group 60–79 years, higher age was associated with more statin use, whereas it was associated with less statin use in the oldest age group. This pattern of age-related statin use was also seen in other studies [12, 15, 21, 32, 33]. Although the most recent guidelines do not recommend age limitations for the use of statins in secondary prevention, current evidence does not support a favorable risk-benefit balance for statins in older persons [32]. Statins have shown to reduce the risk of coronary disease in older persons (70–82 years), but failed to reduce the risk for all-cause mortality and showed a statistically significant 25% increase in incident cancer [52]. Moreover, in the oldest old no trials have been performed and higher cholesterol concentrations have even been associated with longevity in this age group [53]. Furthermore, older age has also been linked to a greater risk of statin adverse effects [50].

The current study showed several comorbidities or cardiovascular risk factors were associated to statin use to a lesser degree. First, hypercholesterolemia was associated with more statin use in secondary prevention. This suggests that physicians prescribe statins as a function of cholesterol levels, despite the fact that statins are recommended in secondary prevention independently of cholesterol level [19, 20, 25, 32]. Second, diabetes predicted greater statin use in the age group 60–79 years. A possible explanation for this is the entrance of a diabetes care program in Belgium, with improved follow-up of these patients and increased awareness of recurrent CVD among patients with diabetes. Furthermore, diabetes has been classified as a coronary heart disease risk equivalent and as a stroke risk equivalent [54]. On the other hand, statin therapy has also been shown to be associated with a slightly increased risk of development of diabetes [55]. Moreover, statins have shown to raise glucose preferentially in patients at risk for diabetes, and to have increased occurrence of adverse effects in patients with diabetes [56]. Third, hypertension was associated with less statin use, possibly because patients and doctors are reluctant to polypharmacy. It has also been shown that geriatric patients of physicians who on average prescribed more medications like cholesterol lowering dugs, had an increased risk of mortality [57]. On the other hand, Xi et al. found that patients with hypertension after stroke are more likely to receive a statin [25]. Furthermore, the ALLHAT-LLT trial showed that pravastatin did not reduce either all-cause mortality or coronary heart disease in older persons with well-controlled hypertension [58]. Fourth, the observed associations between comorbidities like stroke, TIA, dementia and PAD and less statin use could possibly be explained by the higher risk of adverse effects [50, 59–61], the limited effect of statins in people with these conditions [62, 63] and possibly the greater risk of drug errors in people with cognitive problems. Other interesting associations between less statin use and cancer and more statin use and paralysis should be confirmed by further research.

Based on the current findings future research should be organized. First, the effect of a statin treatment in secondary prevention could be estimated in this real world population by using propensity score matching, although this approach is not able to capture key issues of healthy user effects. Second, qualitative research could focus on the reason behind the finding that less than 50% of patients in secondary prevention receive a statin prescription. Although guidelines recommend statins in all age groups, clinicians might be aware of the failure of evidence to support net benefit in key patient groups, including elderly (age > 70 years), or might be reluctant to prescribe statins because of the risk for adverse effects. Furthermore, the impact of patient-related factors and patient preferences should be explored. Third, the current study suggests investigating what factors may be leading to declining CVD risk in older non-statin users.

### Strengths and limitations

The major strengths of the current study are the inclusion of a large real-world study population, representative of the general Flemish population, and the long follow-up period. This study was the first to compute AAPC of the prevalence of statin use in secondary prevention and to examine the prevalence of statin use parallel with the incidence of recurrent CVD in the same population. Furthermore, we were able to examine multivariable models to explain statin use in different age groups and have included a broad range of factors that may be associated with statin use.

However, this study also has limitations. First, this study was an observational study and statin users and non-users could not be considered identical. Therefore, no conclusions could be drawn about the causal effect of statins on the incidence of recurrent CVD. Second, no data were available on mortality. Third, only electronic GP prescriptions were taken into account. Manual prescriptions made during a house visit or prescriptions made by specialists were not included, which might underestimate the real prevalence of statin use. Fourth, we did not investigate the dosage and types of statin patients used and had insufficient data on actual cholesterol levels. Last, no information on smoking status, BMI, race, socio-economic status and time after diagnosis of the first CVD was available. Furthermore, secular trends that may alter CVD risk, like trans fat consumption or treatment of sleep apnea, were not examined.

## Conclusion

The prevalence of statin use in secondary prevention increased strongly from 1999 to 2013, with the increase principally affecting elderly patients. Still, fewer than 50% of the patients with a history of CVD received a statin in 2013. Greater statin use was associated with male sex, medium-older age (increasing up to age 79 years, decreasing thereafter) and diabetes. Persons with cancer, stroke and hypertension were less likely to receive statins. But, the absence of other preventive medications was most strongly correlated with less statin use. The sizable increase in statin use was attended by only a small decline in recurrent cardiovascular events. Moreover, this decline was focused in elderly who were not on statins.

## Abbreviations

AAPC: Average annual percentage change
AIC: AKAIKE information criteria
APC: Annual percentage change
ATC: Anatomical therapeutic chemical
BMI: Body mass index
CI: Confidence interval
COPD: Chronic obstructive pulmonary disease
CVD: Cardiovascular disease
GFR: Glomerular filtration rate
GLMM: Generalized linear mixed models
GP: General practitioner
IHD: Ischaemic heart disease
LDL: Low-density lipoprotein
LLM: Lipid lowering medication
mCCI: Modified Charlson comorbidity index
MDRD: Modification of diet in renal disease
MI: Myocardial infarction
OR: Odds ratio
PAD: Peripheral arterial disease
RAS: Renin-angiotensin system
ROC: Receiver operating characteristic
TIA: Transient ischaemic attack
